# Nonlinear association between changes in fasting plasma glucose and the incidence of diabetes in a nondiabetic Chinese cohort

**DOI:** 10.1186/s12902-022-01094-4

**Published:** 2022-07-27

**Authors:** Chenghu Huang, Chenhong Ren, Xiuping Xuan, Yi Luo, Caibi Peng

**Affiliations:** 1 Department of Endocrinology, Bishan Hospital of Chongqing, Bishan Hospital of Chongqing Medical University, Bishan, Chongqing, 402760 China; 2 Taihe Hospital, Postgraduate Training Basement of Jinzhou Medical University, Hubei University of Medical, Shiyan, 442400 China; 3 Department of Endocrinology, The First Affiliated Hospital of Guangxi Medical University, Nanning, 530021 Guangxi China; 4 Bishan Maternity and Child Hospital of Chongqing, Bishan, Chongqing, 402760 China

**Keywords:** Fasting plasma glucose, Change, Incidence, Diabetes, China

## Abstract

**Background:**

Limited data show that changes in fasting plasma glucose (FPG changes) are related to the incidence of type 2 diabetes (T2D). We aimed to correlate FPG changes with incident diabetes and evaluate FPG changes as a marker to screen participants at high risk of T2D in China.

**Methods:**

A total of 116,816 individuals were followed during a median follow-up of 3.10 years by secondary analysis in a nondiabetic Chinese cohort. The turning points were derived from a receiver operating characteristic curve. Hazard ratios (HRs) were evaluated by Cox proportional hazards models.

**Results:**

A total of 2669 cases of T2D were identified (788 women and 1881 men). The age-standardized incidence of diabetes was 12.87 per 1000 person-years (women: 11.04; men: 14.69). A nonlinear relationship between FPG changes and incident diabetes is shown by the fitting curves. The curves were categorized into three stages by two turning points (-0.04 and 1.25 mmol/L) and conformed to the hook-like pattern: an initial decrease (stage-1), then a transient sharp elevation (stage-2), followed by a slow increase (stage-3). HRs per SD of FPG changes on incident diabetes varied with stage: stage-1: 0.16 (0.12, 0.23), stage-2: 0.20 (0.15, 0.28) and stage-3: 0.22 (0.16, 0.31). Compared with stage-1, the HR in stage-3 was significantly higher at 28.05 (23.99, 32.79), while the increase in stage-2 was slight at 2.16 (1.79, 2.61), and the HR in stage-3 rose to 30.09 (25.02, 36.19).

**Conclusions:**

FPG changes had a strong correlation with the incidence of T2D and was a steady indicator that was used to distinguish the participants at high risk of diabetes.

**Supplementary Information:**

The online version contains supplementary material available at 10.1186/s12902-022-01094-4.

## Background

In recent decades, type 2 diabetes (T2D) has become a large and ever-growing threat and one of the most important public health issues in virtually every region of the world [1]. According to the National Health Interview Survey (NHIS), the prevalence of diagnosed diabetes doubled from 1990 to 2016 (3.5% to 9.7%) [2, 3]. In China, the prevalence of diabetes also increased rapidly from less than 1% in 1980 to 11.2% in 2018 [4]. The threat was consistently underestimated because a significant proportion remained undiagnosed [5–7]. The prediction from the International Diabetes Federation is that 600 million people worldwide will be living with diabetes in 2035 [6, 8]. A high incidence of diabetes-specific complications, such as kidney failure and peripheral artery disease, has a severe impact on quality of life in diabetic patients [9]. Increasingly, diabetes and its complications also bring a heavy burden to patients and the health system [10].

The prevention of diabetes is more important than its treatment. Tracking the evolving epidemiology, it has become clear that the risk factors for T2D include ethnicity, obesity, Western lifestyle, socioeconomic status, prediabetes, and pregnancy [1, 6, 8, 11–13]. Therefore, some traditional risk factors are widely used clinically to predict the occurrence of diabetes, including fasting plasma glucose (FPG), body mass index (BMI), diet, physical activity and metabolic syndrome [14–19]. Among these factors, FPG may be the core predictor of the onset of diabetes [20–22]. In a recent study with 10,796 individuals (aged > 20 years), approximately 70% of prediabetes patients (according to the IFG-ADA: FPG 5.6–6.9 mmol/L) developed T2D within 10 years [23].

Recently, some new metabolic markers, especially variables combined with traditional risk factors, have been found to be closely related to the incidence of T2D.

The triglyceride (TG) to high-density lipoprotein cholesterol (HDL) ratio (TG/HDL ratio) is a good marker to identify insulin resistance in children and adults and is a useful surrogate indicator of future incident type 2 diabetes [24–26]. Another new metabolic marker, the fasting triglyceride-glucose (TyG) index, precedes and significantly predicts T2D [27]. During a median follow-up of 6.17 years in the Tehran Lipid and Glucose Study (TLGS) of urban Iranians, FPG changes was an independent predictor (HR 1.57 [1.31, 1.88]) and the highest quartile of FPG changes increased the T2D risk to 1.65 [1.20, 2.27] compared with the lowest quartile [21]. However, there are limited data on the association between FPG changes and the incidence of diabetes, due to the size of the population and the fact that almost no literature from our region is available on the subject. In our study, we compared these metabolic markers and attempted to develop a simple clinical approach to identify the individuals at greatest risk of incident diabetes by secondary analysis in a nondiabetic Chinese multicenter cohort.

## Material & methods

### Study design and patients

The clinical data were obtained from a public database (https://datadryad.org) and were recorded from January 2010 to December 2016, which was offered by Chen et al. [28]. According to the previous literature, a total of 211,833 subjects were contacted [28, 29]. Briefly, all participants older than 20 years of age were candidates, with complete or valid information, including sociodemographic variables (age and sex), clinical data (height, body weight, body mass index, blood pressure and family history of diabetes), lifestyle data (smoking and alcohol consumption) and biochemical tests (fasting plasma glucose, LDL, HDL, total cholesterol, and triglyceride). In this retrospective cohort study, the following participants were excluded at baseline: those with extreme body mass index (BMI) (< 15 kg/m^2^ or > 55 kg/m^2^), those with shorter visit intervals (< 2 years) and those with diabetes or hypoglycemia at baseline (fasting plasma glucose ≥ 7.0 mmol/L or ≤ 2.8 mmol/L). The diagnosis of incident diabetes was defined as fasting plasma glucose of ≥ 7.00 mmol/L and/or self-reported diabetes during the follow-up period. Moreover, the statistical method boxplot was used to remove 15 outliers of FPG changes. A total of 116,816 participants (53,970 females, 62,846 males) took part in the study. The selection process of the participants was specifically explained previously [28, 29].

### Definitions

The metabolic markers considered were FPG, triglyceride, total cholesterol, and HDL cholesterol, as well as the FPG changes, TyG index and TG/HDL ratio. In our study, FPG changes was defined as the difference between the baseline and final visit FPG (FPG2) (mmol/L) [30]. Participants were excluded with extreme FPG changes (> 15 mmol/L) (*N* = 5). The triglyceride–high-density lipoprotein cholesterol concentration ratio (TG/HDL ratio) was determined by the serum TG (mmol/L): HDL concentration (mmol/L) ratio [^13^]. The triglyceride × fasting plasma glucose (TyG) index was calculated as the natural logarithm (Ln) of [TG (mg/dL) ⁎ glucose (mg/dL)/2] [31].

According to the definition of by the American Diabetes Association (ADA) and the World Health Organization (WHO), fasting plasma glucose (FPG) was analyzed as a categorical variable (< 5.6, 5.6 – 6.0 and 6.1 – 6.9 mmol/L) [7, 32, 33].

Taking into account the effect of the reproductive system on incident diabetes (especially in women), age was analyzed as a ca impaired fasting glucose (IFG)tegorical variable (20–45, 46–55, 56–65 and ≥ 66 years) [34, 35]. As recommended by the Working Group on Obesity in China, normal weight was defined as a BMI of 18.5–23.9 kg/m^2^, overweight as a BMI of 24.0–27.9 kg/m2 and obesity as a BMI of 28.0 kg/m2 or higher [36]. According to the 2010 Chinese guidelines for the management of hypertension, a normal level was defined as systolic blood pressure (SBP) < 120 mmHg and/or diastolic blood pressure (DBP) < 80 mmHg; prehypertension was defined as SBP 120–139 mmHg and DBP 80–89 mmHg; and hypertension was defined as systolic BP (SBP) ≥ 140 mmHg and/or diastolic BP (DBP) ≥ 90 mmHg [37]. Hyperlipidemia was diagnosed according to the Chinese Guidelines for the Management of Dyslipidemia in Adults [38]. Triglyceride (TG) was analyzed as a categorical variable (< 1.7, 1.7—2.2 and ≥ 2.3 mmol/L); total cholesterol (TC) was < 5.2, 5.2 – 6.1 and ≥ 6.2 mmol/L; LDL was < 3.4, 3.4 – 4.0 and ≥ 4.1 mmol/L; and HDL was < 1 and ≥ 1 mmol/L. According to the definition of impaired fasting glucose (IFG) by the American Diabetes Association (ADA) and the World Health Organization (WHO), fasting plasma glucose (FPG) was analyzed as a categorical variable (< 5.6, 5.6 – 6.0 and 6.1 – 6.9 mmol/L) [7, 32, 33].

### Statistical analysis

Data analyses were performed using STATA (Version 13.1, StataCorp LP, College Station, TX, USA). The study data were analyzed for normal distribution, and data with normal and nonnormal distributions were expressed as the mean ± standard deviation (SD) and median (interquartile range), respectively. Time-to-event Cox proportional hazards models were used to assess the hazard ratio (HR) with 95% confidence intervals (CIs) of incident diabetes. The Pearson correlation coefficient is typically used for jointly normally distributed data (data that follow a bivariate normal distribution). For nonnormally distributed continuous data, for ordinal data, or for data with relevant outliers, a Spearman rank correlation can be used as a measure of a monotonic association.

The metabolic markers considered were FPG, triglyceride, total cholesterol and HDL, as well as the FPG changes, TyG index and TG/HDL ratio. Constructing receiver operating characteristic (ROC) curves was used to identify the clinical utility of metabolic markers in the prediction of the onset of diabetes, which depicts the relationship between sensitivity/specificity test results for each metabolic marker [16]. Areas under the ROC curves were compared using the method of Hanley and McNeil [16]. The metabolic marker for predicting the onset of diabetes with the statistically significantly better ROC was selected for turning point analysis to identify specific values by the fitting curves of diabetes probability [16]. We also divided the curves into three different stages by two turning points, and each stage was also divided into three tertiles. For the prospective analyses, we plotted cumulative Kaplan–Meier curves for diabetes development during follow-up. All statistical analyses were performed with the statistical package R (http://www.R-project.org) and EmpowerStats (www.empowerstats.com, X&Y Solutions, Inc., Boston, MA).

## Results

During a median follow-up of 3.10 years (maximum 7.56 years; minimum 2.00 years), 2669 cases of T2D were identified (788 women and 1881 men) among a total of 116,816 participants (53,970 females, 62,846 males). The age-standardized incidence of diabetes was 12.87 per 1000 person-years (women: 11.04; men: 14.69). Men had higher levels of basic clinical data (height, body weight, body mass index, and systolic and diastolic blood pressure), lifestyle data (smoking and alcohol consumption) and biochemical tests (FPG, LDL, total cholesterol and triglyceride) (Table S1). The FPG changes in women was 0.19 ± 0.60 mmol/L lower than that in men (0.25 ± 0.72 mmol/L).

**Fig. 1:**
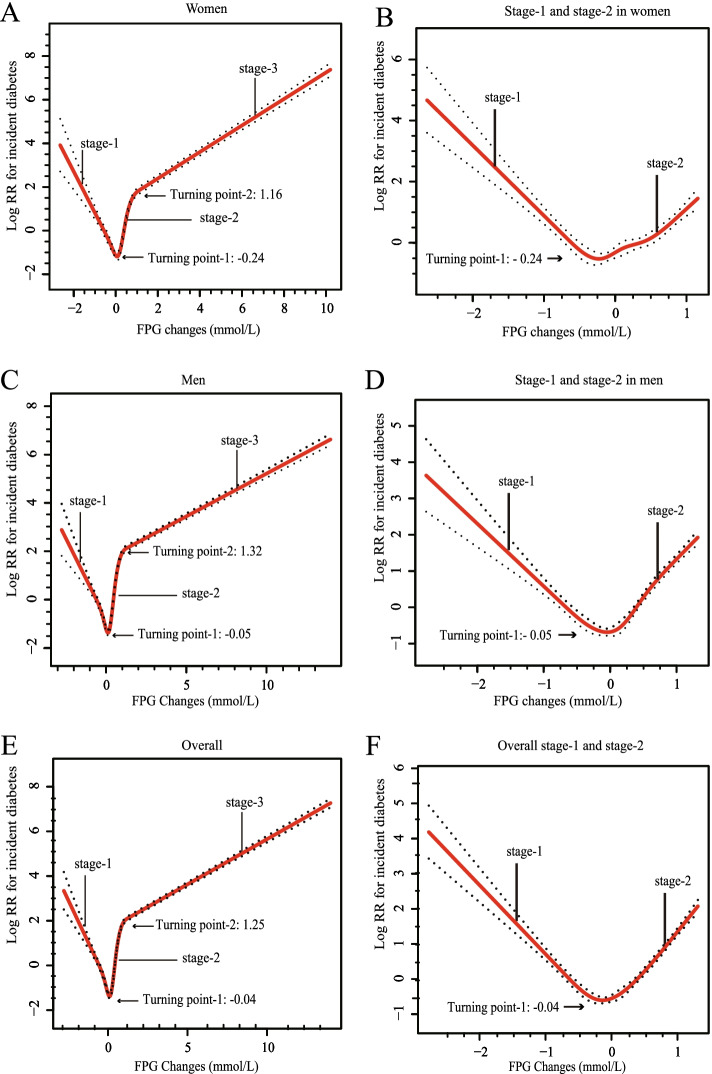
The curves of diabetes probability by FPG changes. FPG changes was defined as the difference between the baseline and final visit FPG (FPG2) (mmol/L). FPG changes were categorized into three stages based on the curves by two turning points

Almost all the fitting curves of diabetes probability of FPG changes were nonlinear and showed a sectional activity that conformed to the hook-like pattern: an initial decrease, then transient sharp elevation, followed by a slow increase. Hence, FPG changes were categorized into three stages based on the curves by two turning points (overall: -0.04 and 1.25; women: -0.05 and 1.16; men: -0.24 and 1.32, mmol/L) (Fig. 1). Importantly, the hook-like pattern was maintained, which was reconstructed via different levels of age, sex, HDL, TG, TC, SBP, DBP and history of diabetes (Fig. S1). Only the curves in participants with LDL ≥ 4.1 mmol/L and/or BMI < 18.5 kg/m^2^ had two stages (Fig. S1). We also found differences in the baseline characteristics of the participants (Table 1). There were similar baseline characteristics in participants with stage-1 and stage-2 FPG changes. However, participants with stage-3 had significantly different basic clinical data (body weight, body mass index, and systolic and diastolic blood pressure) and biochemical tests (LDL, total cholesterol, triglyceride, ALT and AST) in both women and men. Interestingly, from stage-1 to stage-3, the baseline FPG concentration continued to drop and all participants with diabetes in stage-1 were all diagnosed by self-reported (in Table S2). We also found participants with FPG baseline more than 5.6 mmol/L had a higher proportion of diabetics than those with a baseline FPG of less than 5.6 mmol/L both in women and men (in Table S2).

**Table 1:** Baseline characteristic of participants according to sex and stages of FPG changes (mmol/L)

	Women				Men			
	Stage-1 < -0.24	Stage-2 -0.24—1.15	Stage-3 ≥ 1.16	*P*-value	Stage-1 < -0.05	Stage-2 -0.05—1.31	Stage-3 ≥ 1.32	*P*-value
N	12,076	39,132	2762		21,238	38,353	3255	
Age (years)	43.40 ± 12.64	43.40 ± 12.39	48.96 ± 14.26	< 0.001	44.10 ± 13.25	44.26 ± 13.06	48.38 ± 13.64	< 0.001
Height (cm)	160.08 ± 5.64	160.11 ± 5.63	159.20 ± 5.98	< 0.001	171.61 ± 6.26	171.68 ± 6.21	171.49 ± 6.41	0.162
Weight (kg)	56.97 ± 8.24	56.73 ± 8.09	58.50 ± 9.10	< 0.001	71.67 ± 10.64	71.57 ± 10.49	74.41 ± 11.54	< 0.001
BMI (kg/m2)	22.24 ± 3.08	22.13 ± 3.02	23.10 ± 3.51	< 0.001	24.31 ± 3.17	24.26 ± 3.14	25.27 ± 3.45	< 0.001
SBP (mmHg)	115.32 ± 16.37	114.80 ± 16.53	121.72 ± 20.26	< 0.001	123.03 ± 15.60	122.70 ± 15.61	126.15 ± 17.71	< 0.001
DBP (mmHg)	71.48 ± 10.32	71.30 ± 10.42	74.90 ± 11.62	< 0.001	76.69 ± 10.61	76.83 ± 10.71	79.93 ± 11.70	< 0.001
FPG (mmol/L)	5.29 ± 0.47	4.79 ± 0.51	4.42 ± 0.80	< 0.001	5.34 ± 0.50	4.84 ± 0.57	4.62 ± 1.00	< 0.001
TC (mmol/L)	4.81 ± 0.93	4.75 ± 0.91	4.89 ± 0.92	< 0.001	4.84 ± 0.90	4.78 ± 0.87	4.86 ± 0.88	< 0.001
TG (mmol/L)	1.12 ± 0.82	1.07 ± 0.72	1.28 ± 0.90	< 0.001	1.65 ± 1.24	1.59 ± 1.13	1.84 ± 1.31	< 0.001
HDL-C (mmol/L)	1.48 ± 0.31	1.47 ± 0.31	1.47 ± 0.31	0.011	1.29 ± 0.27	1.29 ± 0.28	1.24 ± 0.27	< 0.001
LDL-C (mmol/L)	2.78 ± 0.70	2.73 ± 0.69	2.80 ± 0.67	< 0.001	2.83 ± 0.69	2.77 ± 0.66	2.80 ± 0.65	< 0.001
ALT (IU/L)	17.18 ± 13.82	17.17 ± 14.97	21.00 ± 42.88	< 0.001	28.87 ± 21.55	29.27 ± 24.07	33.83 ± 30.97	< 0.001
AST (IU/L)	21.84 ± 10.48	21.69 ± 9.01	24.44 ± 27.94	< 0.001	25.61 ± 10.60	25.76 ± 13.81	28.39 ± 19.92	< 0.001
BUN (mmol/L)	4.44 ± 1.15	4.37 ± 1.12	4.44 ± 1.18	< 0.001	4.99 ± 1.18	4.91 ± 1.14	4.89 ± 1.16	< 0.001
CCR (μmol/L)	57.82 ± 9.76	58.42 ± 10.51	59.10 ± 11.66	< 0.001	80.43 ± 12.01	80.71 ± 11.97	79.47 ± 11.99	< 0.001
FPG of final visit (mmol/L)	4.76 ± 0.43	5.10 ± 0.48	6.03 ± 1.22	< 0.001	4.93 ± 0.48	5.31 ± 0.55	6.66 ± 1.82	< 0.001
Family history of diabetes, N (%)	355 (2.94%)	1162 (2.97%)	92 (3.33%)	0.533	321 (1.51%)	627 (1.63%)	82 (2.52%)	< 0.001

Continuous variables are presented as the mean ± standard deviation (normal distribution); categorical variables are presented d as number (%). FPG changes was defined as the difference between the baseline and final visit FPG (FPG2) (mmol/L). ALT, alanine aminotransferase; AST, aspartate transaminase; BMI, body mass index; BUN, blood urea nitrogen; CCR, endogenous creatinine clearance rate; DBP, diastolic blood pressure; FPG, fasting plasma glucose; HDL-C, high-density lipoprotein cholesterol; MBP, mean systolic blood pressure; LDL-C, low-density lipoprotein cholesterol; SBP, systolic blood pressure; TC, total cholesterol; TG, triglyceride. ^$^ N (%) refers to the number and proportion of the identical sex group. ^#^ Diagnosis of incident diabetes was defined as fasting plasma glucose of ≥ 7.00 mmol/L and/or self-reported diabetes during the follow-up period. All trend for baseline characteristic of participants among age and groups, *P* < 0.001. In women: stage-1: < -0.24 mmol/L; stage-2: -0.24—1.15 mmol/L; stage-3: ≥ 1.16 mmol/L. In men: stage-1: < -0.05 mmol/L; stage-2: -0.05—1.31 mmol/L; stage-3: ≥ 1.32 mmol/L

Importantly, the incidence of T2D varied with sex and the stages of FPG changes (stage-1: women 1.46, men 2.09, overall, 1.86; stage-2: women 2.14, men 5.59, overall, 3.86; stage-3: women 48.20, men 92.07, overall, 72.45, per 1000 person-years). HR per SD of FPG changes on incident diabetes was also affected by sex and stages of FPG changes. From stage-1 to stage-3, the HRs per SD of FPG changes in women were 0.10 (0.06, 0.18), 4.28 (3.07, 5.98) and 1.56 (1.50, 1.63), respectively; the HRs per SD in men were 0.20 (0.13, 0.30), 8.32 (6.73, 10.29) and 1.27 (1.24, 1.30), respectively; and the overall HRs per SD were 0.16 (0.12, 0.23), 0.20 (0.15, 0.28) and 0.22 (0.16, 0.31), respectively. Compared with stage-1, in women, the HR in stage-3 was significantly higher [22.08 (16.47, 29.59)], while the increase in stage-2 was not significant [1.12 (0.82, 1.51), *P* > 0.05]; in men, the HR in stage-2 slightly increased to 1.81 (1.54, 2.12), and that in stage-3 rose to 30.09 (25.02, 36.19).

**Fig. 2:**
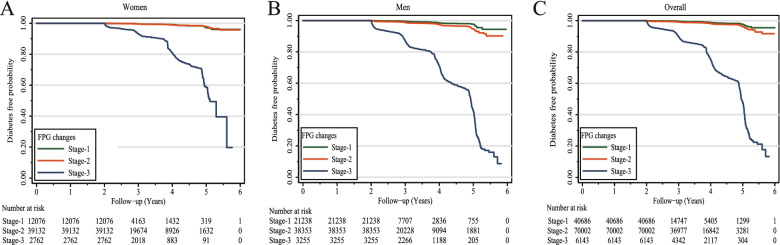
The cumulative diabetes free probability by Kaplan–Meier survival curves in women (**A**), men (**B**) and overall (**C**). FPG changes was defined as the difference between the baseline and final visit FPG (FPG2) (mmol/L). FPG changes was categorized into three stages based on the curves: stage-1: women < -0.24, men < -0.05 and overall < -0.04 mmol/L; stage-2: women -0.24—1.15, men -0.05—1.31 and overall -0.04—1.24 mmol/L; stage-3: women ≥ 1.16, men ≥ 1.32 and overall ≥ 1.25 mmol/L

Kaplan–Meier survival curves for cumulative diabetes-free probability based on sex and the stages of FPG changes are presented in Fig. 2. The cumulative diabetes-free probability was approximately 90% in stage-1 and stage-2 and in both stages at the end of the follow-up period. However, in stage-3, the cumulative diabetes-free probability decreased sharply with the increase in FPG changes, especially in the upper tertile of stage-3, which was close to zero at the end of follow-up (Fig. S2).

We also compared the differences in the correlation between FPG changes and other metabolic markers with the incidence of diabetes by ROC curve analyses (Fig. S3). The area under the curve (AUC) by bootstrap varied with sex: FPG changes 0.864 [0.854, 0.873] (women 0.846; men 0.869), TyG index 0.766 [0.757, 0.774] (women 0.800; men 0.723) and TG/HDL ratio 0.699 [0.690, 0.709] (women 0.740; men 0.646). The predictive model based on FPG changes had better sensitivity (76.83%) and specificity (84.19%) than the model based on the TG/HDL ratio (67.78% and 62.35%, respectively) and TyG index (77.77% and 63.10%, respectively). FPG changes had a higher association with the onset of T2D in both men and women.

## Discussion

The utility of FPG changes in predicting incident diabetes among nondiabetic participants has been reported in a prior study [21]. However, one main finding of this article was that the association between FPG changes and incident diabetes was significant by the secondary analysis in a nondiabetic Chinese multicenter cohort. The association of FPG changes might be stronger than that of other metabolic variables, including TG, FPG, BMI, TG/HDL ratio and TyG index (Fig. S3). The area under the curve (AUC) by bootstrapping for FPG change was highest among these metabolic variables. The sensitivity and specificity of the predictive model based on FPG changes were also higher than those of the TyG index and TG/HDL ratio. The TyG index and TG/HDL ratio were recently suggested as useful diagnostic markers to predict insulin resistance and as predictors for incident diabetes and arterial stiffness [18, 39]. The TyG index and TG/HDL ratio are two reliable alternative markers for insulin resistance in South American overweight and obese children and adolescents [39]. In a 12-year longitudinal study of the Korean Genome and Epidemiology Study cohort, a higher TyG index preceded and significantly predicted T2D among community-dwelling middle-aged and elderly lean Koreans [27]. The advantage of FPG changes are that it might represent the difference between the final and baseline FPG concentrations, and one of the two diagnostic criteria for diabetes in this study is mainly based on FPG at the final visit. In addition, the association between FPG changes and incident diabetes was sex-specific. This specificity may be derived from the differences of the baseline characteristics of the participants and incidence of diabetes between men and women (Table S1 and Fig. 1).

Another glittering prize in this article was that the association of FPG changes with incident diabetes was nonlinear and conformed to a hook-like pattern. The hook-like pattern was a stable stage characteristic, consistent with the distribution of different levels of several variables (including age, sex, TG, SBP, and DBP). In our study, HRs per SD of FPG change on incident diabetes varied according to sex and stage: stage-1: 0.10 (0.06, 0.18) in women and 0.20 (0.13, 0.30) in men; stage-2: 4.28 (3.07, 5.98) in women and 8.32 (6.73, 10.29) in men; stage-3: 1.56 (1.50, 1.63) in women and 1.27 (1.24, 1.30) in men (Table 2). We also found that participants with FPG baseline more than 5.6 mmol/L had a higher proportion of diabetics than those with a baseline FPG of less than 5.6 mmol/L in different stages of FPG changes and different genders (in Table S2). These results are consistent with a large number of epidemiological studies confirming that baseline FPG can be highly predictive of T2D [37, 38]. In a 12-year longitudinal study (ELSA-Brasil), incident diabetes occurred in 5.6%, 22.7% and 53.9% of individuals with FPG < 5.6, 5.6–6.0 and 6.1–6.9 mmol/L, respectively [40]. In the Rotterdam Study with a follow-up of up to 14.7 years, the lifetime risk of progression from prediabetes to T2D was 74.0% in individuals aged 45 years [41].

**Table 2:** The incident diabetes and HR of FPG changes by sex and the stages

	FPG changes		Diabetics, N (%)	Incident Diabetes^#^	HR per SD			HR		
	Stages	Mean ± SD			Crude	Mode1	Mode2	Crude	Mode1	Mode2
Women	Stage 1:	-0.53 ± 0.26	50 (0.41%)	1.46	0.10 (0.06, 0.18)^***^	0.16 (0.09, 0.28)^***^	0.16 (0.09, 0.29)^***^	Ref	Ref	Ref
	Stage 2:	0.31 ± 0.35	263 (0.67%)	2.14	4.28 (3.07, 5.98)^***^	3.47 (2.49, 4.82)^***^	3.24 (2.33, 4.51)^***^	1.12 (0.82, 1.51)	1.13 (0.84, 1.53)	1.19 (0.88, 1.61)
	Stage 3:	1.61 ± 0.70	475 (17.20%)	48.20	1.56 (1.50, 1.63)^***^	1.51 (1.44, 1.58)^***^	1.45 (1.38, 1.52)^***^	22.08 (16.47, 29.59)^***^	15.20 (11.32, 20.41)^***^	14.05 (10.44, 18.89)^***^
Men	Stage 1:	-0.41 ± 0.30	127 (0.60%)	2.09	0.20 (0.13, 0.30)^***^	0.23 (0.15, 0.34)^***^	0.24 (0.16, 0.36)^***^	Ref	Ref	Ref
	Stage 2:	0.46 ± 0.35	684 (1.78%)	5.59	8.32 (6.73, 10.29)^***^	7.65 (6.19, 9.46)^***^	7.19 (5.81, 8.91)^***^	2.16 (1.79, 2.61)^***^	2.15 (1.78, 2.60)^***^	2.17 (1.79, 2.62)^***^
	Stage 3:	2.04 ± 1.23	1070 (32.87%)	92.07	1.27 (1.24, 1.30)^***^	1.29 (1.25, 1.32)^***^	1.22 (1.19, 1.26)^***^	30.09 (25.02, 36.19)^***^	25.58 (21.27, 30.78)^***^	21.95 (18.23, 26.44)^***^
Overall	Stage 1:	-0.46 ± 0.29	177 (0.53%)	1.86	0.16 (0.12, 0.23)^***^	6.91 (5.78, 8.26)^***^	1.31 (1.28, 1.34)^***^	Ref	Ref	Ref
	Stage 2:	0.38 ± 0.36	947 (1.22%)	3.86	0.20 (0.15, 0.28)^***^	6.18 (5.17, 7.38)^***^	1.33 (1.30, 1.36)^***^	1.81 (1.54, 2.12)^***^	1.81 (1.54, 2.13)^***^	1.87 (1.59, 2.19)^***^
	Stage 3:	1.84 ± 1.04	1545 (25.68%)	72.45	0.22 (0.16, 0.31)^***^	5.72 (4.78, 6.84)^***^	1.26 (1.23, 1.29)^***^	28.05 (23.99, 32.79)^***^	22.72 (19.43, 26.58)^***^	19.61 (16.76, 22.96)^***^

Data are odds ratio (95% CI). We calculated odds ratios for type 2 diabetes by multivariable Cox regression analysis. We estimated 95% CIs from FPG (fasting plasma glucose) that represent the amount of information underlying each group (including the reference group). FPG changes was defined as the difference between the baseline and final visit FPG (FPG2) (mmol/L). Mode 1: adjusted for age; Mode 2: adjusted for age, BMI, SBP, DBP, total cholesterol, triglyceride, HDL, LDL and history of diabetes. In women: stage-1: < -0.24 mmol/L; stage-2: -0.24—1.15 mmol/L; stage-3: ≥ 1.16 mmol/L. In men: stage-1: < -0.05 mmol/L; stage-2: -0.05—1.31 mmol/L; stage-3: ≥ 1.32 mmol/L. Overall: stage-1: < -0.04 mmol/L; stage-2: -0.04—1.24 mmol/L; stage-3: ≥ 1.25 mmol/L. ^***^P < 0.001. ^#^ per 1000 persons per year

The strength of our analysis is that the stages of FPG changes, especially stage-3 (FPG changes ≥ 1.16 mmol/L in women and ≥ 1.32 mmol/L in men), could distinguish the participants at higher risk of diabetes. Most of the baseline characteristics, incident diabetes and the HR of FPG changes were significantly higher in stage-3 than in stage-1 and stage-2, while the cumulative diabetes-free probability was markedly lower (Tables 1–2 and Fig. 2).

Our study has several limitations. Firstly, we evaluated the diagnosis of diabetes only by fasting plasma glucose levels in first and final visit. Neither oral glucose tolerance test data nor postprandial plasma glucose levels or glycated hemoglobin measurements of the participants were available to us, which led to underestimation of the incidence of diabetes in final visit. Secondly, the lower the basal fasting blood glucose, the lower the FPG change may be; hence, the prevalence of diabetes might also be lower. Thirdly, there were many missing data on smoking and drinking, so the impact of FPG changes was not adjusted for smoking and drinking. In addition, the participants with FPG changes beyond turning point 2 were very small, less than 10%, which limits the wide application of the research results. Finally, in our study, all participants in stage-1 and most of the participants in stage-2 were diagnosed by self-reported (in Table S2). The diagnosed differences in the proportion of participants with diabetes also influenced the FPG changes. Once diabetes occurs, measures are often taken to control glucose, which leads to a decrease in FPG in final visit.

## Conclusion

We first reported that the correlation between FPG changes and the incidence of T2D was nonlinear by analyzing a nondiabetic Chinese cohort. FPG changes were a steady indicator that could distinguish the participants at higher risk of diabetes (with stage-3 FPG changes).

## Supplementary Information

**Additional file 1: Figure S1.** The curves of diabetes probability by FPG changes in different levels of sex (A), age (B), BMI (C), SBP (D), DBP (E), TC (F), TG (G), LDL (H), HDL (I) and history of diabetes (J). FPG changes was defined as the difference between the baseline and final visit FPG (FPG2) (mmol/L). BMI, body mass index; DBP, diastolic blood pressure; HDL, high-density lipoprotein cholesterol; LDL, low-density lipoprotein cholesterol; SBP, systolic blood pressure; TC, total cholesterol; TG, triglyceride.

**Additional file 2: Figure S2.** The cumulative diabetes free probability by Kaplan-Meier survival curves in women (A), men (B) and overall (C). FPG changes was categorized into three stages based on the curves by two turning points: stage-1: < -0.24 mmol/L in women and < -0.05 mmol/L in men; stage-2: -0.24 - 1.15 women and -0.05 - 1.31 men; stage-3: > 1.16 women and > 1.32 men.

**Additional file 3: Figure S3.** Receiver operating characteristic curves (ROC) of the product of FPG changes, TyG index and TG/HDL ratio for predicting the onset of diabetes in women (A), men (B) and all participants (C). BMI, body mass index; FPG, fasting plasma glucose; FPG changes were defined as the difference between baseline and final visit FPG (FPG2) (mmol/L); The triglyceride–high-density lipoprotein cholesterol concentration ratio (TG/HDL ratio) was counted by serum TG (mmol/L): HDL concentration (mmol/L) ratio; Triglyceride × fasting plasma glucose (TyG) index was calculated as the natural logarithm (Ln) of [TG (mg/dL) ⁎ glucose (mg/dL)/2].

**Additional file 4: Table S1.** Baseline characteristic of participants according to sex.

**Additional file 5: Supplemental Table 2.** The proportion of diagnosed diabetics in different stages of FPG changes and different genders.

## Data Availability

The clinical data and materials were obtained from a public database (https://doi.org/10.5061/dryad.ft8750v).

## Abbreviations

ALT: Alanine aminotransferase
AST: Aspartate transaminase
AUC: Area under the curve
BMI: Body mass index
BUN: Blood urea nitrogen
CCR: Endogenous creatinine clearance rate
DBP: Diastolic blood pressure
FPG: Fasting plasma glucose
FPG changes: Changes in fasting plasma glucose
HDL-C: High-density lipoprotein cholesterol
HR: Hazard ratios
IFG: Impaired fasting glucose
MBP: Mean systolic blood pressure
LDL-C: Low-density lipoprotein cholesterol
ROC: Receiver operating characteristic
SBP: Systolic blood pressure; T2D: type 2 diabetes
TG/HDL ratio: Triglyceride to high-density lipoprotein cholesterol ratio
TC: Total cholesterol
TG: Triglyceride
TyG: Triglyceride-glucose
