# Supplementary material for: Nonlinear association between changes in fasting plasma glucose and the incidence of diabetes in a nondiabetic Chinese cohort
Source: BMC Endocr Disord. 2022 Jul 27;22:191. doi: 10.1186/s12902-022-01094-4 (PMC9327176; doi:10.1186/s12902-022-01094-4)
Supplement: Supplementary file 4 — Additional file 4: Table S1. Baseline characteristic of participants according to sex. [file 12902_2022_1094_MOESM4_ESM.docx]

Table S1 Baseline characteristic of participants according to sex

|  | Women | Men | P-value^*^ |
| --- | --- | --- | --- |
| N | 53970 | 62846 |  |
| Age (years) | 43.68 ± 12.61 | 44.42 ± 13.19 | <0.001 |
| Height (cm) | 160.06 ± 5.65 | 171.65 ± 6.24 | <0.001 |
| Weight (kg) | 56.87 ± 8.19 | 71.75 ± 10.62 | <0.001 |
| BMI (kg/m^2^) | 22.21 ± 3.07 | 24.33 ± 3.17 | <0.001 |
| SBP (mmHg) | 115.27 ± 16.77 | 122.99 ± 15.74 | <0.001 |
| DBP (mmHg) | 71.52 ± 10.49 | 76.94 ± 10.76 | <0.001 |
| FPG (mmol/L) | 4.88 ± 0.57 | 5.00 ± 0.63 | <0.001 |
| TC (mmol/L) | 4.77 ± 0.92 | 4.81 ± 0.88 | <0.001 |
| TG (mmol/L) | 1.09 ± 0.75 | 1.63 ± 1.18 | <0.001 |
| HDL-C (mmol/L) | 1.47 ± 0.31 | 1.29 ± 0.28 | <0.001 |
| LDL-C (mmol/L) | 2.74 ± 0.69 | 2.79 ± 0.67 | <0.001 |
| ALT (IU/L) | 17.37 ± 17.31 | 29.37 ± 23.69 | <0.001 |
| AST (IU/L) | 21.86 ± 11.17 | 25.85 ± 13.27 | <0.001 |
| BUN(mmol/L) | 4.39 ± 1.13 | 4.94 ± 1.16 | <0.001 |
| CCR (μmol/L) | 58.32 ± 10.41 | 80.55 ± 11.99 | <0.001 |
| FPG of final visit(mmol/L) | 5.07 ± 0.60 | 5.25 ± 0.76 | <0.001 |
| Years of follow-up | 3.10 ± 0.94 | 3.10 ± 0.95 | 0.502 |
| Diabetes, N (%) |  |  | - |
| No | 53182 (98.54%) | 60965 (97.01%) |  |
| Yes | 788 (1.46%) | 1881 (2.99%) |  |
| Smoking status, N (%)^$^ |  |  | <0.001 |
| Current smoking | 17(0.03%) | 6654(12.33%) |  |
| Ever smoking | 11(0.02%) | 1317(2.44%) |  |
| Never smoking | 11409(21.14%) | 13275(24.59%) |  |
| Not recorded | 42540(78.82%) | 41608(77.09%) |  |
| Drinking status, N (%)^$^ |  |  | <0.001 |
| Current drinking | 16 (0.03%) | 861 (1.60%) |  |
| Ever drinking | 310 (0.57%) | 5224 (9.68%) |  |
| Never drinking | 11108 (20.58%) | 15157 (28.08%) |  |
| Not recorded | 42528(78.81%) | 41594(77.09%) |  |
| History of diabetes (yes), N (%)^$^ |  |  | <0.001 |
| No | 52361 (97.02%) | 61816 (98.36%) |  |
| Yes | 1609 (2.98%) | 1030 (1.64%) |  |
| FPG changes (mmol/L) | 0.19 ± 0.60 | 0.25 ± 0.72 | <0.001 |
| TG/HDLC | 0.80 ± 0.69 | 1.37 ± 1.14 | <0.001 |
| TyG | 8.20 ± 0.56 | 8.60 ± 0.60 | <0.001 |

Continuous variables are presented as the mean ± standard deviation (normal distribution); categorical variables are presented d as number (%). ALT, alanine aminotransferase; AST, aspartate transaminase; BMI, body mass index; BUN, blood urea nitrogen; CCR, endogenous creatinine clearance rate; DBP, diastolic blood pressure; FPG, fasting plasma glucose; HDL-C, high-density lipoprotein cholesterol; MBP, mean systolic blood pressure; LDL-C, low-density lipoprotein cholesterol; SBP, systolic blood pressure; TC, total cholesterol; TG, triglyceride.

FPG changes were defined as the difference between baseline and final visit FPG (FPG2) (mmol/L). The triglyceride–high-density lipoprotein cholesterol concentration ratio (TG/HDL ratio) was counted by serum TG (mmol/L): HDL concentration (mmol/L) ratio. Triglyceride × fasting plasma glucose (TyG) index was calculated as the natural logarithm (Ln) of [TG (mg/dL) ⁎ glucose (mg/dL)/2].

^$^ n (%) refers to the number and proportion of the identical sex group. ^#^ Diagnosis of incident diabetes was defined as fasting plasma glucose of ≥7.00 mmol/L and/or self-reported diabetes during the follow-up period. All trend for baseline characteristic of participants among age and groups, P <0.001.
