# Supplementary material for: Nonlinear association between changes in fasting plasma glucose and the incidence of diabetes in a nondiabetic Chinese cohort
Source: BMC Endocr Disord. 2022 Jul 27;22:191. doi: 10.1186/s12902-022-01094-4 (PMC9327176; doi:10.1186/s12902-022-01094-4)
Supplement: Supplementary file 5 — Additional file 5: Supplemental Table 2. The proportion of diagnosed diabetics in different stages of FPG changes and different genders. [file 12902_2022_1094_MOESM5_ESM.docx]

Supplemental Table 2. The proportion of diagnosed diabetics in different stages of FPG changes and different genders

|  | FPG changes (mmol/L) | | Diabetics, N (%) ^#*^ | | | Diabetics by self-reported, N (%) ^*^ | | |
| --- | --- | --- | --- | --- | --- | --- | --- | --- |
|  | Stages | Range | Overall | FPG at baseline (mmol/L) | | Overall | FPG at baseline (mmol/L) | |
|  |  |  |  | < 5.6 | 5.6-6.9 |  | < 5.6 | 5.6-6.9 |
| Women | Stage 1 | < -0.24 | 50 (0.41%) | 7 (0.07%) | 43 (1.65%) | 50 (0.41%) | 7 (0.07%) | 43 (1.65%) |
|  | Stage 2 | -0.24 - 1.15 | 263 (0.67%) | 54 (0.15%) | 20 (15.41%) | 141 (0.37%) | 54 (0.15%) | 87 (6.42%) |
|  | Stage 3 | ≥ 1.16 | 475 (17.20%) | 214 (8.59%) | 261 (95.96%) | 128 (4.63%) | 65 (2.61%) | 63 (23.16%) |
| Men | Stage 1 | < - 0.05 | 127 (0.60%) | 18 (0.11%) | 109 (1.95%) | 127 (0.60%) | 18 (0.11%) | 109 (1.95%) |
|  | Stage 2 | -0.05 - 1.32 | 684 (1.78%) | 77 (0.22%) | 607 (19.08%) | 232 (0.60%) | 77 (0.22%) | 155 (4.87%) |
|  | Stage 3 | ≥ 1.32 | 1070 (32.87%) | 440 (16.76%) | 630 (100%) | 630 (6.02%) | 79 (3.01%) | 117 (18.57%) |
| Overall | Stage 1 | < -0.04 | 199 (0.49%) | 31 (0.10%) | 168 (1.89%) | 199 (0.49%) | 31 (0.10%) | 168 (1.89%) |
|  | Stage 2 | -0.04 - 1.24 | 891 (1.27%) | 125 (0.19%) | 766 (16.62%) | 349 (0.50%) | 125 (0.19%) | 224 (4.86%) |
|  | Stage 3 | ≥ 1.25 | 1579 (25.77%) | 654 (12.59%) | 925 (99.04%) | 326 (5.32%) | 144 ( 2.77%) | 182 (19.49%) |

FPG changes was defined as the difference between the baseline and final visit FPG (FPG2) (mmol/L). FPG, fasting plasma glucose. ^*^ N (%) refers to the number and proportion of the identical sex and stage group. ^#^ Diagnosis of incident diabetes was defined as fasting plasma glucose of ≥7.00 mmol/L and/or self-reported diabetes during the follow-up period.
